# Age-related effects of executive function on takeover performance in automated driving

**DOI:** 10.1038/s41598-022-08522-4

**Published:** 2022-03-30

**Authors:** Qijia Peng, Yanbin Wu, Nan Qie, Sunao Iwaki

**Affiliations:** 1 Graduate School of Comprehensive Human Sciences, University of Tsukuba, Tsukuba, Japan; 2 Human-Centered Mobility Research Center (HCMRC), National Institute of Advanced Industrial Science and Technology (AIST), Tsukuba, Japan; 3 Department of Industrial Engineering, Tsinghua University, Beijing, People’s Republic of China; 4 Present Address: Human Informatics and Interaction Research Institute (HIIRI), National Institute of Advanced Industrial Science and Technology (AIST), Tsukuba, Japan

**Keywords:** Human behaviour, Risk factors

## Abstract

The development of highly automated vehicles can meet elderly drivers’ mobility needs; however, worse driving performance after a takeover request (TOR) is frequently found, especially regarding non-driving related tasks (NDRTs). This study aims to detect the correlation between takeover performance and underlying cognitive factors comprising a set of higher order cognitive processes including executive functions. Thirty-five young and 35 elderly participants were tested by computerized cognitive tasks and simulated driving tasks to evaluate their executive functions and takeover performance. Performance of n-back tasks, Simon tasks, and task switching were used to evaluate updating, inhibition, and shifting components of executive functions by principal component analysis. The performance of lane changing after TOR was measured using the standard deviation of the steering wheel angle and minimum time-to-collision (TTC). Differences between age groups and NDRT engagement were assessed by two-way mixed analysis of variance. Older participants had significantly lower executive function ability and were less stable and more conservative when engaged in NDRT. Furthermore, a significant correlation between executive function and lateral driving stability was found. These findings highlight the interaction between age-related differences in executive functions and takeover performance; thus, provide implications for designing driver screening tests or human–machine interfaces.

## Introduction

In 2021, the population aged 60 years and older was over 1 billion worldwide, and one in six people will be 60 years or older by 2030^1^. In ageing societies, the proportion of elderly drivers are rapidly increasing. In Japan, one of the countries with the highest proportion of elderly citizens, 29.1% of the population was over 65 years old in 2021^2^, which will be 37.7% by 2050^3^. Moreover, the proportion of people over 70 years old with a driver’s license reached 14.5% in 2019, increasing by approximately 15 times during the past 30 years^4^.

Driving is an important way for the elderly to maintain their mobility, which is known to strongly affect their independence and quality of life^5,6^. However, elderly drivers are more likely to be involved in crashes at intersections, lane changing, and driving accidents involving multiple vehicles^7–9^. Some elderly people even have to give up driving due to the decline in their physical and mental conditions^10–12^.

The development of highly automated vehicles, also known as level 3 automation defined by SAE International^13^, is expected to benefit elderly drivers by enhancing their mobility^14–16^. During level 3 automated driving, drivers can engage in non-driving related tasks (NDRTs); while in certain situations (e.g., an unexpected construction zone or broken vehicle on a highway) when the highly automated system fails and prompts a takeover request (TOR), e.g. an auditory signal, drivers must be able to regain control of the vehicle. Elderly drivers tend to engage in various NDRTs during simulated automated driving^17^, including entertainment, working, and dietary activities^18^.

### Effects of NDRTs on takeover performance

Engaging in NDRTs is known to affect takeover performance, such as more collisions^19^ and more deviation from the center of the lane^20^. The negative effect of NDRT is especially crucial for elderly drivers, considering the decline in their driving ability. Therefore, elderly drivers’ behaviors after a TOR could be more cautious or conservative than younger drivers. For example, they prefer to brake more and maintain a longer safe distance^17,21,22^. Moreover, slower reactions and decision making among elderly drivers were found when regaining control after a TOR^23^. Moreover, after a period of manual driving, an increase in reaction time (RT) to a TOR was found for elderly drivers, but not for younger drivers^24^. Furthermore, Wu et al.^25^ revealed age-related differences in the interaction between NDRT and drowsiness and found that performing NDRT did not alleviate the drowsiness of elderly drivers but deteriorated their takeover performance. The distribution of visual attention can also differ between young and elderly drivers. That is, elderly drivers focused more on the road than NDRT compared with younger drivers^26^. Moreover, they checked their back mirrors less frequently and their fixation time became shorter when engaging in NDRT^27^.

Other studies reported no significant age effects on driving performance after a TOR^22^ or takeover time^28^. Despite the different study designs, these contradictory results suggest that other factors may underlie age-related differences. Li et al.^29^ investigated takeover performance, behavior, and perceptions of elderly people in different age subgroups, suggesting that elderly people should be considered as a heterogeneous group. Beyond chronological age, it is important to explore individual differences in driving performance in response to TOR using explicit factors.

### Executive function and elderly drivers’ driving behaviors

Driving is a complex task that requires various cognitive abilities. For instance, visual attention and visuospatial cognition are related to overall driving performance^30,31^: perceptual-cognitive capacity is related to speed control and crash risk^32^, simple RT is related to hazard perception^33^, and working memory is related to decision making^34^. These cognitive abilities are associated with age^35,36^; therefore, elderly drivers with worse cognitive abilities are more likely to exhibit dangerous driving behaviors and be involved in crashes^37–39^.

Adrian et al.^40^ concluded that functional abilities were more determinant than chronological age in predicting elderly drivers’ performance. Studies have shown that age-related decline in various cognitive tasks could be attributed to the aging of the frontal lobe, also known as the frontal aging hypothesis^41,42^. Furthermore, the frontal lobe has been demonstrated to be correlated with executive function (EF)^43,44^. Executive functions comprises a set of higher-order cognitive processes that coordinate lower-level processes^45^, and can be defined as processes that control and regulate thought and action^46^. Executive functions may play a crucial role in driving tasks because it requires complex cognition-required task sets to summarize information and supervise actions^47^, such as maintaining continued attention, dealing with irrelevant information, and adapting to different task requirements^48^.

Three main components of executive functions were distinguished: “Shifting,” “Updating,” and “Inhibition”^49^. The shifting component refers to shifting between multiple tasks, operations, and mental sets^50^. The updating component involves dealing with incoming information to replace the old, already irrelevant information^51^. The inhibition component can be described as an active or willed suppression of the tendency to react to more dominant or automatic responses^49,52^.

Previous research has revealed an important relationship between driving performance and executive functions. Daigneault et al. found that elderly drivers with lower executive abilities were more likely to be involved in traffic accidents^47^. More specifically, correlations between the three executive components and driving performance have been demonstrated in previous studies. To illustrate, shifting ability (mostly measured by trail making tasks) was found to be correlated with car crash risk^37^, road test driving behaviors^30^, hazard perceptions^33^, and driving errors in lane position^53^. Researchers also found that inhibition ability, which is related to selective attention, was associated with road test scores^54^, on-road driving performance^31^, and observation errors^53^. Updating ability, which is related to working memory^55^, plays an important role in drivers’ decision making^34^.

In comprehensive studies dedicated to the three main components of executive functions, updating^40,56^, inhibition^57^, and shifting^40^ components were found to be significantly correlated with driving performance. Walshe et al.^48^ reviewed various subprocesses of executive functions and concluded that inhibition and updating played important roles in car crashes. Moreover, executive function components resulting from principal component analysis (PCA)^56^ and confirmatory factor analysis^57^ showed clear correlations between these latent factors and driving performance. The use of underlying, structured components yielded from the results of cognitive tests rather than pure task performance can alleviate the complexity of executive functions research brought on by the so-called “task impurity problem.” Executive tasks often involve other cognitive tasks; therefore, executive function test results may provide additional individual differences unrelated to executive functions^49^. Exploring the correlation between driving performance and executive function components as latent variables can avoid these uncertainties and produce more valid results^57^. Therefore, we expected that the use of latent components generated from executive functions task performance could effectively establish a correlation with takeover performance.

Overall, several studies have focused on age-related differences in takeover performance, as well as the importance of abilities related to executive functions in driving behaviors. However, research on the relationship between age-related differences in executive function components and takeover performance in automated driving is rare. To fill this gap, we designed a series of computerized cognitive tests and simulated driving tasks to evaluate drivers’ executive functions and takeover performance. Executive function components were extracted by PCA and their correlations with takeover performance were investigated. The purpose of this exploratory study is two-fold: first, to examine whether age-related differences exist in executive functions and takeover performance in automated driving, and second, to explore the relationship between specific executive function components and takeover performance.

## Methodology

### Participants

Participants who were active licensed drivers and in normal health were recruited from the local community (Tsukuba City, Japan). We used opt-in recruitment method: online and offline recruitments were advertised in local community, and totally 70 participants signed up for this study. Thirty-five of them were elderly drivers above 65 years (M = 72.8 years, SD = 3.6 years), including 17 males and 18 females. Furthermore, 35 were younger drivers below 35 years (M = 27.8 years, SD = 4.4 years), including 24 males and 11 females. The sex ratio did not differ significantly between groups (chi-squared = 2.12, df = 1, p = 0.145). All participants provided informed consent complying with the research protocol approved by the Institutional Review Board of the National Institute of Advanced Industrial Science and Technology (AIST). Ninety percent of the participants drove more than 1,000 km per year (34% = 1,000–5,000 km per year; 58% = more than 5,000 km), and drove frequently in daily life (30% drove 3–4 days per week, and 59% drove daily). 54.3% of the participants indicated that driving was their only mean of daily travel, and 20% of the participants indicated that 75% of their daily travel was driving. No significant difference of these proportions between age groups were found.

### Procedure

Before the experiment, the experimenter welcomed and introduced the participants to the experiment purpose and contents. Participants were then introduced to three cognitive tasks. Prior to each task, participants were given detailed task instructions and sufficient training. After completing the cognitive tasks, participants were given a 10-min break. Hereafter, they were introduced to the simulator-based driving tasks. Participants were provided with two driving practice sessions: a manual driving session with simple maneuvers (e.g., lane changing), and an automated driving session with TOR scenarios similar to that used in the formal experiment. After familiarizing themselves with the simulator operation and driving task requirements, participants completed the experimental driving session.

### Materials

The driving task was conducted on a set of desktop driving simulators at the AIST. The driving simulator apparatus included a set of Logitech steering wheel and pedals as input devices, a Panasonic screen (87 mm × 154 mm) with an approximate 45-degree field-of-view for visual presentation, and a set of Mitsubishi Driving Simulator software packages for monitoring and recording.

### Tasks for executive functions

**Figure 1:**
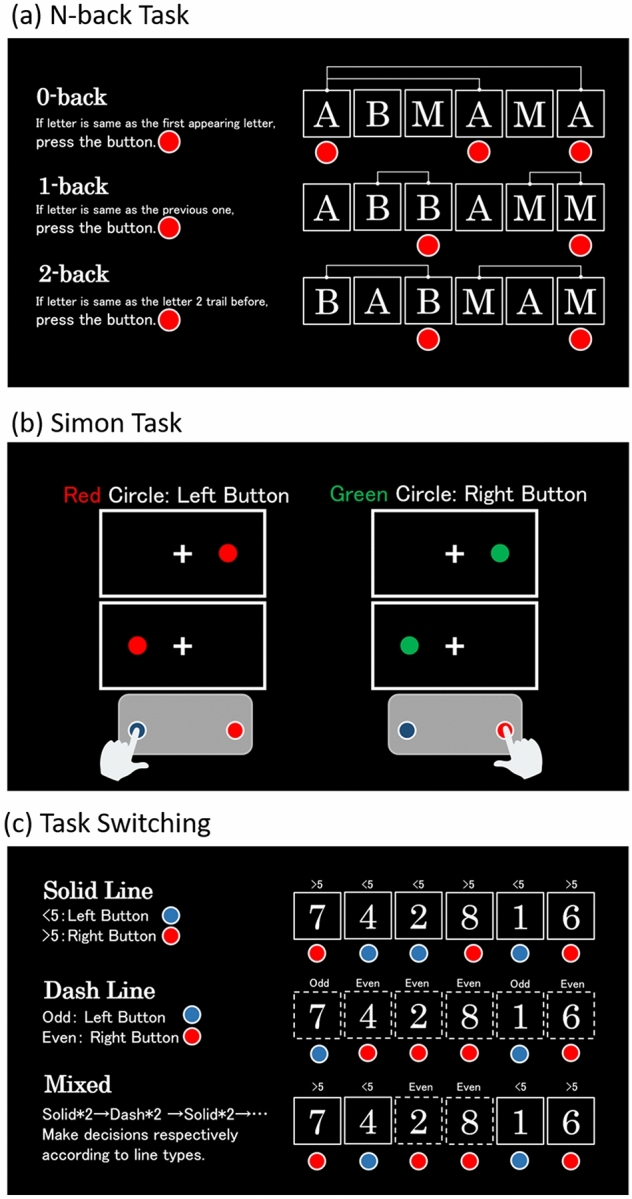
Illustration of three cognitive tasks. Participants were given detailed instructions before each task. All instructions were shown in Japanese during actual experiments.

All participants were asked to complete three computerized tasks on a laptop, measuring the executive functions (EF) components. For all tasks, participants observed visual stimuli shown on a laptop screen running Psychopy software and responded only to target stimuli by pressing previously specified buttons on the keyboard. Each task lasted 12–15 min, with a 5-min break between tasks. Illustrations of each task are shown in Fig. 1.

#### Updating

This component was assessed using a classic version of the sequential letter n-back task^56,58–60^.

In the 0-back condition, the target was the first letter of sequence in the block; in the 1-back condition, the target was any letter identical to the letter immediately preceding it; in the 2-back condition, the target was any letter identical to the letter appearing two trials before. Letters in uppercase appeared in random order for 500 ms with a 2,000 ms interval before the onset of the next trial.

Participants completed 12 trials in each block and 12 blocks in total. Before each block, the task type of this block was shown at the center of the screen. Each block and trial were presented in a randomized order.

#### Inhibition

This component was assessed using a visual version of the Simon task paradigm^61,62^. Each block began with a fixation cross at the center of the screen. In each trial, a red or green circle appeared on the left or right side of the cross and remained visible for 500 ms. Participants were instructed to press the left button when they saw a green circle, and the right button when they saw a red circle. After the offset of the stimulus, there was a 2,000 ms interval before the onset of the next trial.

Participants completed 12 trials in each block and 12 blocks in total. Six of the blocks were congruent conditions, consisting of congruent trials in which the stimuli were presented on the same side as the associated response button. The other six blocks were mixed conditions, in which each block consisted of half congruent and half incongruent trials (stimuli were presented on the opposite side as the associated button). Each block and trial were presented in a randomized order.

#### Shifting

This component was assessed using a variant digit version of the task switching paradigm^63–65^.

This paradigm included two digital-related tasks. In each trial, a numeric digit (from 1 to 9, excluding 5) was presented at the center of the screen and was surrounded by a solid or a dashed square. The task rules were determined as follows: if the square was solid-lined, participants needed to determine whether the digit was greater or less than 5; if the square was dash-lined, they needed to determine whether the digit was odd or even. Participants were instructed to press two buttons on the keyboard according to different responses. Digits were presented for 1,500 ms in each trial with a 2,000 ms interval before the onset of the next trial.

Participants completed 12 trials in each block and 16 blocks in total. Eight blocks were task homogeneous conditions, in which only one type of task would appear, and the other eight blocks were task heterogeneous conditions, in which two types were mixed and each type of task would take turns to appear twice. The choice for alternating runs was believed to increase participants’ working memory demands when they tried to keep track of the tasks; thus, was a more sensitive way to detect group differences^64,66^. Each block and trial were presented in a randomized order.

### Driving experiment

A 2 (age) × 2 (NDRT) between- and within-subjects mixed factor experiment was designed. The between-subjects factor was age (young and elderly drivers), and the within-subject factors were NDRT engagement (no additional task and task engaged).

#### Driving task scenario

**Figure 2:**
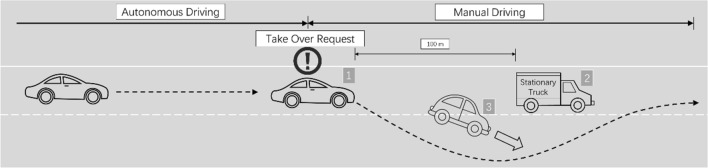
Scenario of takeover request and driving tasks.

Participants encountered a variant version of the Lane Changing Task^67^ after TOR in a simulated level 3 automated vehicle. Similar scenarios in the automated driving version are commonly used to evaluate drivers’ takeover performance when engaging with NDRT^17,24,68^. In this study, we chose a typical scenario: a lane-blocking stationary truck was suddenly revealed by the lane changing of the front car (see Fig. 2).

The participants in the self-driving car (vehicle 1) were in automated driving most of the time, until warned by an audio warning message “Transition to manual driving” in Japanese as the TOR and were then asked to take over full control (both steering wheel and pedals). Participants had to change lanes to avoid collision and pass the stationary truck (vehicle 2), which was revealed by the lane changing of a leading car (vehicle 3). The speed set for automated driving and all moving vehicles were 25 m/s. The TOR occurred at 100 m in front of the stationary truck. Moving cars (every 50 m per vehicle) were set in the right lane next to the participant’s current lane, so the participants also had to pay attention to avoid collisions. The scenario ended when the participant drove 200 m after the stationary truck, also noted by an audio notice of “Automated driving starts”.

Based on previous research on the time budget or lead time for takeover, the common choices were in the range between 4.5 and 7.5 s^17,24,69^. In this study, we set time budget to 4 s to make the scenario riskier and put the participants under more pressure.

#### Non-driving related task

The NDRT during autonomous driving was chosen as a sequential-letter 0-back task. For every 2 s, the voice of a letter was randomly played by the speaker, and participants were required to press a button on the steering wheel if they heard the target letter. The NDRTs continued for approximately 40 trials until the TOR occurred, and ended when the TOR audio was played.

The auditory, but not visual, NDRT was designed to avoid additional visual distraction in the traffic environment. Based on our pilot study, the current version of the auditory 0-back task of moderate difficulty was chosen to keep participants (especially elderly participants) involved in the NDRT during automated driving.

### Measurements

#### Executive function components

The measures for the working memory updating component were RTs for each correct response and accuracy in each condition in the n-back task. For the inhibition component, RTs for each correct response and accuracy in both congruent and mixed blocks were recorded, and the Simon effect^70^ was calculated based on the difference in RTs between congruent and incongruent trials in mixed blocks in the Simon task. For the shifting component, RTs for each correct response, accuracy in task homogeneous and task heterogeneous conditions, and switch cost^71^ were calculated by the difference in RTs between trials after switching and non-switching trials in task heterogeneous conditions in the task-switching task.

#### Driving performance

Driving performance data were recorded at a rate of 60 Hz. Raw data included position, speed, and acceleration data of the vehicle, as well as driver input data (e.g., steering wheel and pedal operations).

According to similar studies^24,25^, we chose two proven valid measures to evaluate the quality of takeover performance:

- sdSteer: standard deviation of the steering wheel angle, calculated by the standard deviation of the steering wheel angle after TOR before the end of manual driving. This indicated the ability to regain control of the vehicle and smooth lane change.
- TTC: time-to-collision calculated by the distance at the moment of the lane change of the vehicle divided by the instant velocity. This indicated the ability to maintain a longitudinal safety margin when changing lanes in response to a TOR.

## Results

### Executive functions evaluation

#### N-back performance

Main effects of age group were found by a two-way mixed ANOVA: the young group performed faster in RTs (F(1,68) = 7.82, p = 0.007) and had higher accuracy (F(1,68) = 26.87, p < 0.001) than the elderly group. The increase in task complexity also showed a significant main effect on the increase in RTs (F(2,136) = 111.77, p < 0.001) and a decrease in accuracy (F(2,136) = 113.26, p < 0.001). Moreover, significant age group × task complexity interactions were observed for RTs (F(2,136) = 7.83, p < 0.001) and accuracy (F(2,136) = 24.16, p < 0.001).

The simple effect test demonstrated that for RTs, in the 0-back (t = 1.19, df = 63.46, p = 0.240) and 1-back (t = 1.68, df = 67.67, p = 0.097) tasks, there were no significant differences between age groups, while the elderly group had significantly longer RTs (t = 3.55, df = 64.81, p < 0.001). For response accuracy, there was no significant difference between age groups in the 0-back (t = −0.27, df = 67.48, p = 0.785) task, but there were significant losses in accuracy for the elderly group in the 1-back (t = −2.81, df = 50.87, p = 0.007) and 2-back (t = −6.36, df = 58.67, p < 0.001) tasks.

#### Simon task performance

We found main effects between age groups in which younger participants performed faster in RTs (F(1,68) = 15.71, p < 0.001) than the elderly; however, no significant difference in accuracy (F(1,68) = 1.27, p = 0.264) between age groups was found. We also detected a significant increase in RTs (F(1,68) = 16.59, p < 0.001) and a decrease in accuracy (F(1,68) = 4.57, p = 0.036) in incongruent compared to congruent trials. However, no significant interaction was found for RTs (F(1,68) = 1.13, p = 0.292) or accuracy (F(1,68) = 3.43, p = 0.068). Moreover, no significant difference in the Simon effect was found (t = 1.06, df = 50.55, p = 0.293) between the two age groups.

#### Task switching performance

We found main effects between age groups where younger participants had faster RTs (F(1,68) = 28.73, p < 0.001) and higher accuracy (F(1,68) = 10.56, p = 0.002) than the elderly. The main effect of task switching was also detected; in switch trials, participants showed longer RTs (F(1,68) = 369.99, p < 0.001) and less accuracy (F(1,68) = 28.27, p < 0.001). Furthermore, there were two-way interactions in the RTs between the age groups and switch conditions (F(1,68) = 11.87, p < 0.001). This can be explained by a significantly larger difference in RTs between switch and non-switch trials (the switch cost, also shown in Table 1) found in the elderly group than in the younger group (t = 3.45, df = 53.8, p = 0.001). The descriptive statistics for EF tasks are presented in Table 1.

**Table 1:** Descriptive statistics for executive function tasks.

Task	Condition	Reaction time (ms)		Accuracy (%)	
		Elder	Young	Elder	Young
n-back	0-back	481.6 ± 15.6	458.5 ± 11.8	96.9 ± 0.9	97.3 ± 0.8
	1-back	547.0 ± 19.1	503.1 ± 17.8	92.9 ± 1.4	97.3 ± 0.7
	2-back	727.7 ± 27.4	603.5 ± 21.8	75.0 ± 1.9	89.7 ± 1.3
Simon task	Congruent	671.6 ± 23.1	580.0 ± 15.5	94.7 ± 1.4	97.5 ± 1.1
	Incongruent	705.9 ± 18.3	600.1 ± 14.8	94.5 ± 1.1	97.3 ± 1.2
	Simon Effect	34.3 ± 11.9	20.1 ± 6.1	–	–
Task switching	Non-switch	900.9 ± 21.6	783.8 ± 19.1	94.3 ± 1.0	97.6 ± 0.5
	Switch	1124.7 ± 22.9	939.7 ± 20.5	90.0 ± 1.4	95.0 ± 0.9
	Switch cost	224.0 ± 17.2	155.9 ± 9.7	–	–

Data are presented as mean ± standard deviation. The Simon effect and switch cost were only calculated by RT.

#### Principal component analysis for executive function components

PCA was conducted based on the measures of cognitive tasks in order to generate more reasonable, structured components representing the abilities of executive functions, and thus to show clearer correlation with takeover behaviors.

The subject to item ratio in our study is 7.78:1 (70 participants in total and 9 measures from different tasks), which is larger than the commonly recommended minimum subject to item ratio of 5:1^72^.The PCA results of the selected measures of performance in EF tasks (normalized data) are shown in Table 2. The measures comprised RTs and accuracy results for the tasks. Accuracy in the 0-back condition was not selected because the differences between individuals and groups were not significant. The measures for RTs in the Simon task and task switching was chosen as the Simon effect and switch cost in each task. The loadings for each component are presented in Table 2. The principal components were ordered by their percent of variance explained.

**Table 2:** Loadings for the principal component analysis of the executive function data.

	PC1	PC2	PC3	PC4	PC5	PC6	PC7	PC8	PC9
0-back RT	**0.45**	− 0.35	0.10	− 0.17	0.20	− 0.28	0.19	− 0.02	0.70
1-back RT	**0.45**	− 0.22	0.32	− 0.19	0.08	0.13	0.29	− 0.45	− 0.55
2-back RT	**0.46**	− 0.30	− 0.07	0.11	0.16	0.25	− 0.44	0.59	− 0.21
1-back Accuracy	− 0.22	**− 0.50**	− 0.48	− 0.13	0.17	− 0.13	− 0.44	− 0.46	− 0.07
2-back Accuracy	− 0.38	**− 0.51**	− 0.03	− 0.14	− 0.04	− 0.28	0.48	0.45	− 0.26
Simon Task Accuracy (incongruent trials)	− 0.08	− 0.28	**0.58**	0.36	− 0.45	− 0.33	− 0.37	− 0.09	0.00
Simon Effect	0.12	− 0.10	− 0.28	**0.86**	0.16	− 0.04	0.32	− 0.14	− 0.03
Task Switching Accuracy (switching trials)	− 0.36	− 0.34	0.30	0.10	0.14	0.74	0.07	− 0.07	0.27
Switch Cost	0.22	− 0.17	− 0.38	− 0.07	**− 0.81**	0.28	0.15	− 0.04	0.11
Eigenvalue	2.75	1.42	1.34	1.03	0.87	0.57	0.41	0.32	0.30
Percent of variance (%)	30.58	15.77	14.89	11.43	9.63	6.31	4.57	3.52	3.30
Cumulative percent of variance (%)	30.58	46.35	61.24	72.67	82.30	88.61	93.18	96.70	100.00

The loadings of main compositions were marked bold.

The results showed that PC1–PC4 had eigenvalues greater than one. As for PC5, despite the eigenvalue of 0.87, the difference of percentage of variance explained between PC4 (11.4%) and PC5 (9.6%) was not evident, yet the decrease between PC5 and PC6 (6.3% of variance, 0.57 of eigenvalue) was sharp. Considering the percentage of variance explained, PC4 and PC5 both accounted for about 10% of the variance individually, and by including PC5 there were over 80% (82.30%) of cumulated variance. Therefore, we chose the first 5 components in percent of variance and yielded a five-component solution for further analyses. The interpretation of the main composition of each principal component and the EF components is shown in Table 3.

**Table 3:** Main compositions and classification of each component of the PCA.

	Reaction speed related	Accuracy related
Updating	PC1: RTs in n-back tasks	PC2: Accuracy in n-back tasks
Inhibition	PC4: Simon Effect	PC3: Accuracy in Simon tasks
Shifting	PC5: Switch Cost	–

### Driving performance

**Figure 3:**
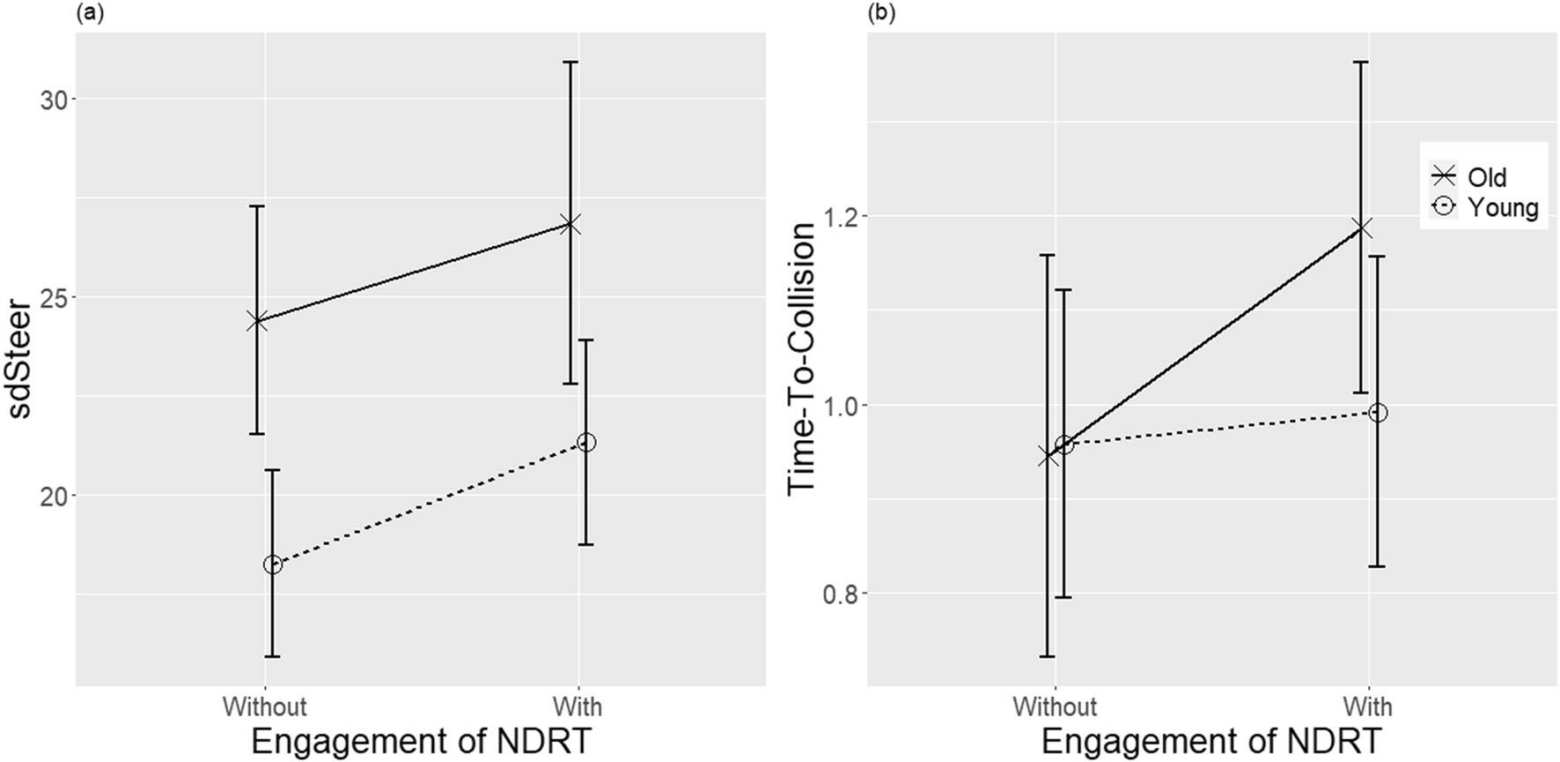
Differences of driving performance between age groups and the conditions of NDRT engagement, measured by (**a**) steering wheel standard deviation and (**b**) time-to-collision. NDRT: non-driving related tasks.

Driving performance was evaluated by the standard deviation of the steering wheel angle (sdSteer) during the manual driving period, and the TTC at the moment of the lane change. Outliers were identified as observation points outside 1.5 * Inter Quartile Range, which was the difference between the 75th and 25th quartiles of the dataset. The observations higher and lower than the boundary were replaced by the higher and lower limits of the values of the 95th and 5th percentiles of the dataset. The differences between the groups and conditions are presented in Fig. 3.

#### Standard deviations of steering wheel angle (sdSteer)

The results of the two-way mixed ANOVA showed that age had a main effect on sdSteer, with the elderly group (M = 25.62, SD = 10.08) having significantly larger sdSteer than the young group (M = 19.78, SD = 7.31; F(1,67) = 10.72, p = 0.002). The main effect was also found between conditions with and without the NDRT, where sdSteer was higher in conditions with NDRT (M = 24.04, SD = 10.09) than without (M = 21.28, SD = 8.12; F(1,67) = 6.12, p = 0.016). No significant interaction was found between age and engagement in NDRT (F(1,67) = 0.07, p = 0.788).

#### Time-to-collision

The results of the two-way mixed ANOVA revealed no significant main effect in conditions with and without engagement in NDRT, with longer TTC when engaged (M = 1.09, SD = 0.50) than when not engaged (M = 0.95, SD = 0.54), although the difference was not statistically significant (F(1,67) = 2.97, p = 0.089). No significant main effect was found in the age groups (F(1,67) = 0.90, p = 0.347) and no significant interaction was found between age and engagement in NDRT (F(1,67) = 1.72, p = 0.194).

### Correlations between executive function components and driving performance

**Figure 4:**
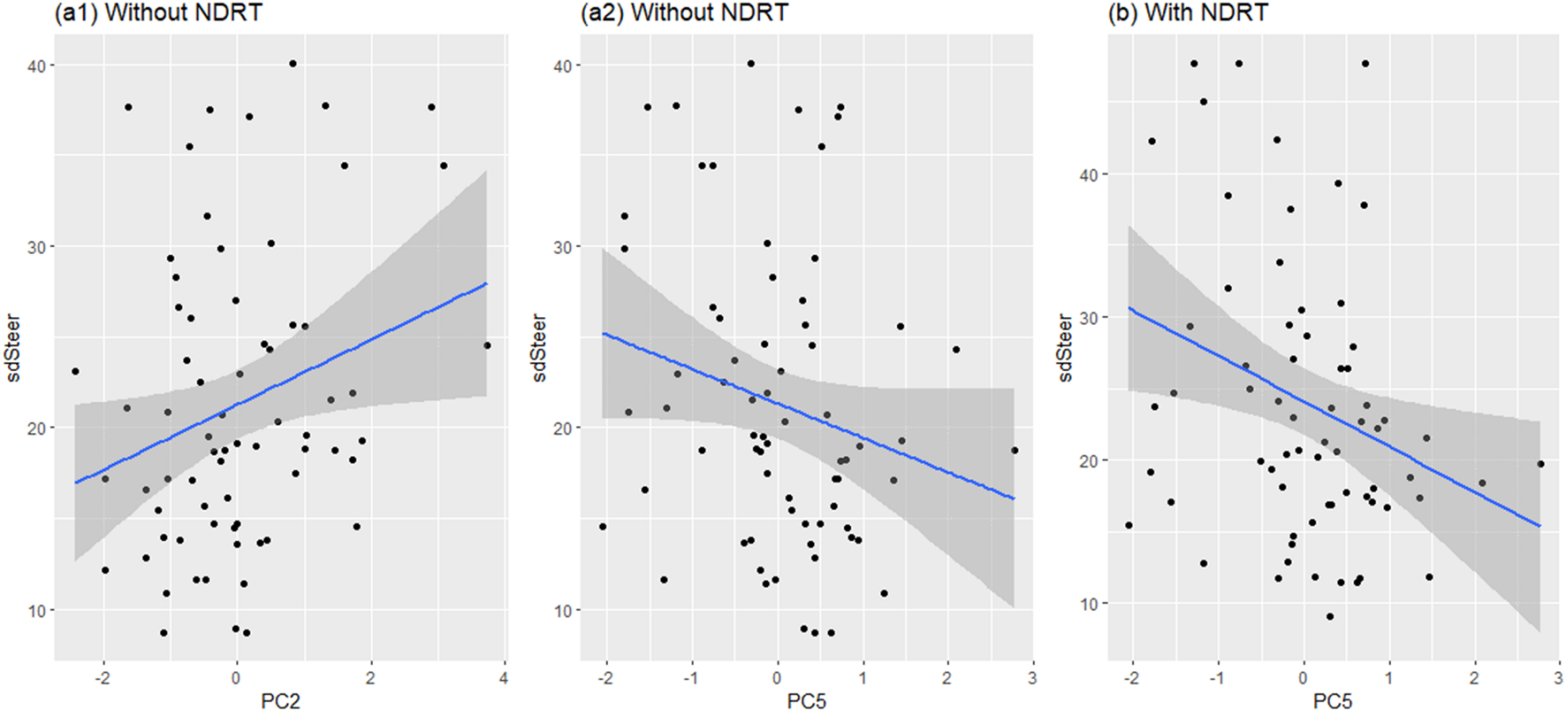
Scatterplots showing the correlations of standard deviations of steering wheels (sdSteer) and different components of (**a1**) PC2 without NDRT, (**a2**) PC5 without NDRT, and (**b**) PC5 with NDRT. Blue lines indicate linearly fitted smooth lines, and shades around lines present 95% confidence intervals. PC2: principal component 2, PC5: principal component 5, NDRT: non-driving related tasks.

Correlations between EF components (results from the PCA of EF measures) and driving performance (sdSteer and TTC) were analyzed for each condition (with/without engagement in NDRT) using Pearson’s correlation coefficients (see Fig. 4). Correlations were calculated on data from all participants of both groups in each condition.

A significant correlation was found between sdSteer and accuracy of the updating component (PC2, r = 0.26, p = 0.029) without NDRT engagement, and a sdSteer tended to correlated with the cost of shifting component (PC5, r = −0.22, p = 0.075), although the correlation was not statistically significant. Moreover, no significant correlation was found between TTC and EF components.

When engaging in NDRT, sdSteer was significantly correlated with the cost of the shifting component (PC5, r = −0.29, p = 0.015); no significant correlation was found between TTC and EF components.

## Discussion

This study investigated age-related differences in the effects of NDRT on takeover performance and examined how individual differences in specific components of executive functions could influence takeover performance during automated driving.

### Executive functions and takeover performance

#### Executive function performance

The results of executive functions tasks showed that younger participants had better performance in both reaction speed and accuracy in most conditions of all tasks, indicating that they had better working memory updating, inhibition control, and mental shifting abilities. The age-related differences were consistent with those of previous studies on executive functions^73,74^. Results of accuracy failed to show significant differences between age groups in some conditions, such as the 0-back, 1-back, and congruent Simon task. This may have been because the task could have been too easy for participants in both age groups to detect differences in results. Surprisingly, we did not find a significant difference in the Simon effect, which was larger in the elderly group than in the young group in similar studies^62^. However, in our study, the individual difference in the Simon effect was large within the elderly group. Considering that the accuracy for Simon tasks was relatively high, even for the elderly group, we believe that our Simon task was not difficult enough to detect differences in inhibition control, but mainly reflected components about choice RT.

Moreover, the PCA results of the cognitive tasks data yielded a construct of three main executive function components: working memory updating, inhibition control, and mental shifting. This result was consistent with previous research^40,56,57^, suggesting that these three executive function components are separable, while the cognitive tasks could be correlated with one another^49^. The use of PCA-yielded components rather than pure task performance measures for further analysis can assure orthogonality of these components representing different parts of executive functions. Furthermore, our construct clearly distinguished between reaction speed (PC1, PC4) and accuracy (PC2, PC3) related components in both updating and inhibition; this could contribute to a detailed analysis of the role speed and accuracy played in the correlation between each executive function component and driving performance.

#### Driving performance

In the analysis of differences in driving performance between age groups and engagement in NDRT, we found significantly less stable lateral maneuvering in elderly drivers (p = 0.002) engaging in NDRT (p = 0.016). These findings are consistent with previous studies on the influence of age^18,25^ and NDRT^17,20^ on drivers’ takeover performance. Moreover, the NDRT used in most previous studies required visual attention, while the NDRT in this study (i.e., n-back task) mainly required auditory attention, indicating that pure cognitive workload could significantly influence takeover performance^19,75^.

In the analysis of the TTC at the moment of lane change, we found no significant difference between age groups; however, participants tended to have longer TTC when engaging in NDRT, although the effect was not statistically significant (p = 0.089). The results of the number of participants who stepped on the acceleration pedal after the TOR showed that younger drivers (15 out of 35) tended to accelerate when engaged in NDRT compared to elderly drivers (6 out of 35). This suggests that the higher TTC in our study may be explained by the conservative behaviors of elderly drivers dealing with a TOR when engaged in NDRT. Similarly, previous studies found that elderly drivers in similar scenarios tend to exercise more caution and drive slower^17,22^, or apply more frequent and stronger brake to maintain a longer TTC^21^ after transitioning to manual driving.

### Correlations between executive function components and driving performance

The main finding in this study was that better ability to update and shift components was found to be significantly correlated with more stable lateral driving performance after takeover. The shifting component was found to be important for lateral control in both situations, regardless of engagement in NDRT. Similar results were also found in previous studies^40^, wherein the shifting ability measured by the score of the plus-minus task was significantly correlated with on-road driving performance. Moreover, a better shifting ability is considered to reflect drivers’ performance in switching between the NDRT and driving tasks in urgent situations. A tendency of shifting components to affect driving stability were detected even without engaging in NDRT although the correlation was not statistically significant. This indicated that shifting ability may play a more important role in a takeover task, compared with other driving tasks mentioned in previous studies, such as manual driving lane changing^56^. This could be partly explained by the complexity of a takeover task: it requires the driver to shift more frequently and urgently between different task sets. Furthermore, previous studies have detected a relationship between driving behaviors and shifting ability in a trail making test^30,40,76^, wherein the task may contain both cognitive shifting (involving visual attention) and task shifting components. In this study, both the task for evaluating shifting ability (variant digit version of task switching) and the NDRT (audio 0-back task) did not require a shift in visual attention. Thus, even without visual attention shifting, mental shifting ability influenced drivers’ performance in responding to the TOR.

The updating component was also significantly correlated with a more stable driving performance, which is consistent with previous research^40,56^. This significant correlation with the working memory updating accuracy was found in situations without NDRT. For a takeover driving task, the content of working memory must be updated and irrelevant information deleted; therefore, poor updating retains irrelevant information and reduces the processing of relevant information^40^. Thus, when encountered with a situation requiring takeover, drivers with poor updating ability could not process the incoming information (such as speed and position status for both self-driving and other vehicles) very well, which led to less stable driving performance. Another important finding was that a significant correlation with the accuracy component of working memory updating was found. In our study, the design of the driving task left a relatively sufficient time budget for the drivers to take over. This showed that the accuracy of updating (ability to update correct information) compared with the speed of updating (ability to update swiftly), may be more important for lateral stability in a takeover task.

Previous research has demonstrated the contribution of inhibition components to driving performance^57^. In addition, selective attention, which is related to inhibition ability, was found to be linked to driving performance^31,53^. However, this study did not detect a significant correlation between inhibition components and driving performance under any condition; this finding was still consistent with a previous similar study in which various executive function components were involved^40,56^. There may be two possible reasons for this inconsistent finding. First, our design of an easy Simon task may have led to poor discrimination of inhibition ability. That is, we found no significant difference in the Simon effect between age groups; thus, it may be difficult to detect sufficient individual differences in inhibition ability by using the Simon task performance. Second, in our experiment scenarios, the priority of NDRT was lower than taking over the vehicle; thus, the NDRT might have been considered less crucial than the driving task by the participants. That is, as our measures for the inhibition component focused on active suppression for a dominant response, takeover performance could be less relevant to this measure if the NDRT was not prepotent over the takeover task. In such case, the variance of takeover performance may be more related to individual differences in shifting rather than inhibition ability.

We detected a significant difference in TTC between age groups but found no significant correlation between executive function components and TTC. Although the TTC reflects the safety margin in responding to the TOR, it could also be influenced by drivers’ driving style and strategy in urgent situations rather than executive functions. That is, other non-EF-related factors, such as personality and attitudes^77^, may affect the driving behaviors of elderly drivers.

### Application

Our findings contribute to a better understanding of drivers with age-related differences in executive functions interacting with a TOR in highly automated vehicles. Knowledge of the relationship between individual differences in executive functions and driving behavior can be used to evaluate driving capability. Research on general assessment, such as the trace-route task method, also showed that it could be a valid and reliable tool for evaluating executive functions and distinguishing drivers with risky driving behaviors^78^. Similarly, our findings can guide quick screening for elderly drivers’ cognitive status for a TOR of highly automated vehicles, such as an exam for elderly drivers’ license updating, or a lite system monitoring the cognitive status of elderly drivers. Moreover, an understanding of this relationship could help human–machine interface (HMI) design to support elderly drivers by considering their executive function status. Previous studies also suggested that age-related changes in cognitive functions should be considered in the design and application of automated driving for elderly drivers^79^, and the perceived usability of HMI design is related to cognitive performance^80^. This study’s findings can further provide more information about drivers’ behaviors and cognitive status. Thus, these results can benefit HMI design, especially for drivers with age-related declines in executive functions.

### Limitations

While the study revealed the possible correlation between executive function components and driving performance after a TOR, there were several limitations that must be discussed.

First, the use of a desktop simulator may have limited our ability to discover valid correlations. A previous study found that the association of executive function components with driving could be mediated by computer game skills, and the relations were even stronger when the skilled participants were excluded^56^. Thus, future research should utilize more real-world driving simulators with higher fidelity to investigate driving behaviors. Second, due to the design of the NDRT, participants answered the NDRT by pressing buttons on the steering wheel, which means that the RTs after the TOR could have been difficult to measure precisely. Since executive functions may be highly correlated with RTs, further investigation on the correlation between RT-related measures and executive function ability is needed. Third, the task evaluating inhibition control was not effective enough to reveal individual differences and to yield significant results for a correlation with driving performance. Therefore, further research is necessary to build credible correlations between driving performance and inhibition ability using more valid cognitive measures.

## Conclusion

In this study, we designed and conducted a series of computerized cognitive and simulated driving tasks to evaluate executive functions and takeover performance. We examined (1) the executive function components of updating, inhibition, and shifting yielded by results from cognitive tasks, and (2) takeover performance of lateral maneuvering by steering wheel standard deviations and longitudinal safety margin by the TTC at the moment of lane change. The results demonstrated a significantly lower ability of executive functions among elderly participants and less stable and more conservative driving behavior of elderly drivers when engaged in NDRT. We further observed a significant correlation between executive functions (working memory updating accuracy and shifting cost) and takeover performance. Overall, these findings provide important insights into elderly drivers’ driving behaviors and executive functions, which can be utilized to expand on the present study’s findings and improve driving safety for elderly.

## Data Availability

The data are available on request from the corresponding author upon reasonable request.
